# The proposed modification of TNM staging and therapeutic strategy for skip metastasis in papillary thyroid carcinoma: A multicenter retrospective cohort study

**DOI:** 10.1002/cam4.6018

**Published:** 2023-05-04

**Authors:** Zheyu Yang, Yu Heng, Qiwu Zhao, Ding Hao, Lei Tao, Xiaxing Deng, Wei Cai, Weihua Qiu

**Affiliations:** ^1^ Department of General Surgery Ruijin Hospital, Shanghai Jiaotong University School of Medicine Shanghai China; ^2^ Department of Otolaryngology Eye Ear Nose & Throat Hospital, Fudan University Shanghai China; ^3^ Department of General Surgery Civil Aviation Shanghai Hospital Shanghai China

**Keywords:** lymph node metastasis, papillary thyroid carcinoma, recurrence, skip metastasis, TNM staging

## Abstract

**Background:**

Skip metastasis is a special type of lateral lymph node metastasis, which is not classified definitely by the eighth edition of the AJCC TNM staging system. The aim of the research was to study the prognosis of skip metastasis in PTC patients, and carry out a more appropriate N staging for skip metastasis.

**Methods:**

Study subjects were 3167 patients with papillary thyroid carcinoma (PTC), who underwent thyroidectomy at three clinical centers from 2016 to 2019. We identified two well‐balanced cohorts matched on the basis of propensity score.

**Results:**

During a median follow‐up of 42 months, recurrence occurred in 68 (4.3%) patients with lymph node metastasis. 34 cases recurred in 1120 patients with central lymph node metastasis (N1a), and 34 recurred in 461 patients with lateral lymph node metastasis (N1b), among which 73 patients were diagnosis with skip metastasis. The RFS of N1a was significantly lower than that of N1b (*p* < 0.001). After propensity‐score matching, recurrence rate was significantly lower in the skip metastasis group than in the LLNM group (*p* = 0.039), whereas the rate was similar in the skip metastasis groups and the CLNM group (*p* = 0.29).

**Conclusions:**

In conclusion, our study indicated that, among patients with LLNM, those with positive skip metastasis showed significantly lower recurrence, exhibiting a similar rucurrence tendency as patients with CLNM. Thus, skip metastasis could be categorized into N1a stage rather than N1b stage based on the AJCC TNM staging system. The downstaging of skip metastasis may reveal more conservative treatment strategy.

## Abstract 2

Our study provided the largest number of skip metastasis cases among current studies. Our series of studies on skip metastasis have progressed step by step, from the characteristics of primary tumor site to the possible factors of skip metas occurrence leading to the correlation between skip metas and prognosis in the present study. We proposed adjustments to the current AJCC TNM staging system. We provided potential treatment strategy for skip metas in PTC patients.

**Lay summary**

This study analyzed 3167 patients from three clinical centers that underwent thyroidectomy for PTC within the period 2016–2019.We focused on skip metastasis, a class of lymph node metastases with a low incidence based on our previous systematic researches (Heng et al. Front Endocrinol, 2021; Yang et al. Front Endocrinol, 2021). The study indicated that, among patients with LLNM, those with positive skip metastasis showed significantly lower recurrence, exhibiting a similar recurrence tendency as patients with CLNM. Thus, skip metastasis could be categorized into N1a stage rather than N1b stage based on the AJCC TNM staging system. The downstaging of skip metastasis may reveal more conservative treatment strategy.

## 1 INTRODUCTION

Papillary thyroid carcinoma (PTC), the most common type of endocrine malignancy with rapidly increasing incidence, but stable high survival rate of exceeding 98 percent in 5‐year follow‐up.^1^, ^2^, ^3^ Given that the excellent survival rate and long follow‐up time of PTC, the routine criteria to determine prognosis of cancer patients may not be appropriate for PTC. Several articles have mentioned that the ability to determine the risk of disease recurrence may be a more meaningful outcome for patients and clinicians than the risk of disease‐specific death.^4^, ^5^, ^6^ The 2015 American Thyroid Association (ATA)‐modified initial risk stratification system (RSS) classified recurrence risk as low, intermediate, or high, and defined various risk factors, in which lymph node metastasis (LNM) played an important role.^7^, ^8^

Regional LNM occur first in the paratracheal and prelaryngeal/tracheal lymph nodes of the central compartment and subsequently in the lateral compartment.^9^, ^10^ According to the American Joint Committee on Cancer (AJCC, 8th edition) staging system, LNM is divided into N1a (levels VI, VII) and N1b (levels I, II, III, IV, or V).^11^ Skip metastasis, leaping over central compartment, is a specific type of lateral cervial metastasis, which is routinely classified as N1b. Despite both 8th AJCC and 2015 ATA‐RSS combined N1a and N1b into N1 for staging and stratification, treatment and follow‐up strategies for N1a staging are significantly different from those for N1b, of which have larger surgical area, more postoperative complications, and higher recurrence risk.^12^, ^13^, ^14^ The 8th AJCC reclassified level VII lymph node compartment metastasis from N1b staging to N1a staging, indicating that accurate staging of lymph node metastasis is the key point to treat PTC patients. When further researching N staging based on our clinical data, we found that skip metastasis, a particular type of lymph node metastasis, presented clinical characteristics inconsistent with the AJCC staging system. We tend to conduct an accurate staging of skip metastasis, providing a more individualized and minimally invasive treatment strategy for this special group of PTC patients.

In recent studies, clinicians and researchers have a more positive attitude about the less extensive surgeries, which parallel the underlying theme of “less is more” presented in the 2015 ATA guidelines.^15^, ^16^, ^17^ This trend embodies a core concept important in surgery: under the pretext of radical resection of the tumor, the preservation of thyroid glands and functions should be maximized, along with minimizing complications to ensure patients' quality of life. The present study, we reclassified skip metastasis from the original N1b classification, which not only provided a more accurate classification standard, but also provided the possibility of less surgical intervention for PTC patients with skip metastasis.

## 2 MATERIALS AND METHODS

### 2.1 Patients selection and exclusion

A retrospectively collected, multicenter database from Shanghai, China was analyzed over a 5‐year period for patients who underwent first‐time thyroidectomy for PTC. Clinical centers involved in the study included Department of General Surgery, Ruijin Hospital, Shanghai Jiao Tong University School of Medicine, ENT Institute and Department of Otorhinolaryngology, Eye and ENT Hospital of Fudan University and Department of General Surgery, Civil Aviation Shanghai Hospital. A total of 3814 patients were enrolled in the study. The exclusion criteria were as follows: (1) no follow‐up or unavailable follow‐up data (n = 511); (2) no histologically proven PTC (*n* = 90) or histologically proven poorly differentiated papillary cancer (PDTC) (*n* = 24), (3) no lymph nodes removed (*n* = 23), (4) history or coexistence of other head and neck cancer (*n* = 6), (5) initial diagnosis staging T_x_N_x_M_1_ (*n* = 16). After exclusion, 3167 patients that had pathological PTC, and received thyroidectomy between January 2016 and March 2019 were studied for enough follow‐up duration.

### 2.2 Data sources and surgical procedures

Preoperative data sources including serum index, ultrasound examination (US) and fine‐needle aspiration (FNA) were collected from Electronic Medical Records System for further analysis. Serum index examination included blood routine, liver and renal function, electrolyte, DIC, and thyroid function in all enrolled patients, CEA and other tumor markers, parathyroid function in some patients. Preoperative US and US‐guided FNA were performed strictly according to Thyroid Imaging Reporting and Data System (TI‐RADS).^18^ In addition to thyroid and parathyroid glands, description of central and lateral lymph nodes were also included.

According to 2015 Tumor Node Metastasis (TNM) staging system of American Joint Committee on Cancer (AJCC), 8th edition, all patients enrolled were identified as T_0‐4_N_0‐1b_M_0_. Surgery was performed by thyroid specialists of high operation volume at Ruijin Hospital, Civil Aviation Shanghai Hospital and Eye and ENT Hospital.^19^, ^20^ Specifically, total thyroidectomy was performed by experienced surgeons with the help of nerve monitoring and intraoperative carbon nanoparticles. Surgical procedures include total thyroidectomy and thyroid lobectomy with routine central compartment lymph node dissection (LND), and lateral LND including ipsilateral levels IIa, III, IV, VI in patients with lateral LNM.

All acquired specimens were examined by two or more board‐certified pathologists from Shanghai Ruijin Hospital and Shanghai EENT Hospital. Pathological features analyzed were pathological type of tumor, type of the surrounding thyroid tissues, tumor size, multifocality (more than one lesion in unilateral thyroid lobe), and lymph node metastasis.

### 2.3 Establishing criteria for propensity‐score matching

Propensity‐score matching (PSM) has been widely used in the clinical research due to its advantage of minimal bias in comparative studies. A retrospectively maintained database parameters from our previous experience and other studies was searched to conduct the case‐match analysis.^21^ The database included information on patient characteristics, disease processes, postoperative and follow‐up records. In total, 73 cases of skip metastasis, 388 cases of LLNM and 1120 cases of central lymph node metastasis (CLNM) were included in our study. We established a propensity score for each patient through logistic regression modeling and then patients were matched 1:1, with the caliper width set as 0.01 for the SD. Standardized mean differences were estimated before and after matching to evaluate the balance and a value less than 0.1 was considered not significant between treatment groups. Age, sex, body mass index (BMI), hypertension history, diabetes history, diffuse thyroid disease, Hashimoto's thyroiditis, nodular goiter, bilateral lesions, capsular invasion, maximum tumor diameter, and multifocality were selected as covariates. Finally, 62 of 388 patients with LLNM, 59 of 1120 patients with CLNM were matched with those with skip metastasis, respectively, with the aim of minimizing bias of the pairwise comparison between the three groups: LLNM Group, CLNM Group, and Skip Group.

### 2.4 Follow‐up and criteria for recurrence

Patients who underwent surgery were assessed for perioperative and postoperative short‐term complications during hospitalization and recorded in the progress notes. Patients enrolled were treated with postoperative TSH suppression therapy and RAI (Radioactive Iodine) therapy according to the 2015 ATA Guidelines. Patients were followed up regularly while the first, second, and third follow‐up were scheduled in the first, second,and third months after the operation, and the follow‐up visits were made at intervals of 6 months. Follow‐up methods included outpatient records, electronic medical record (EMR), and telephone, online follow‐up. Thyroid function examination including thyroglobulin (Tg) and other serum index were reexamined within half a year after operation, while US together with serum examination were performed every 6 months starting from half an year after operation.

In this study, recurrence, excluding new lesions in residual thyroid gland, included recurrence in thyroidectomy bed, lymph nodes, and distant site. Recurrence was defined as structural recurrence after completion of initial treatment, identified using imaging modalities, that is, US examination and/or radioactive iodine‐131 (RAI) whole‐body scan imaging, followed by cytological or histological confirmation, regardless of serum levels of Tg.

### 2.5 Ethical statement

Written informed consent was obtained and the research was approved by the local Ethics Committee and the Institutional Review Board, including the Institutional Ethics Committee of the Eye and ENT Hospital of Fudan University, Ruijin Hospital, Shanghai Jiao Tong University School of Medicine, and Civil Aviation Shanghai Hospital, and was also approved by Chinese Clinical Trial (ChiCTR2100043353). All patients were fully informed of the experimental procedures and patient data including demographics, operative procedures, pathology, and complications, were retrospectively collected.

### 2.6 Statistical analysis

Chi‐square and Student's t‐test were conducted for comparing categorical and continuous variables of baseline and clinicopathological characteristics, respectively. Kaplan–Meier method and log‐rank test were used to compare recurrence‐free survival estimates. All the above‐mentioned statistical analyses were conducted using the SPSS 24.0 package (SPSS Inc.), and a two‐sided *p*‐value <0.05 was considered as statistical significance. Hazard ratio and the relative 95% confidence interval (CI) were also calculated. Propensity‐score matching (PSM) was used to balance the patients' characteristics, with the aim of collecting bias of the pairwise comparison between the three groups: LLNM Group, CLNM Group, and Skip Group. A caliper width of 0.1 of standard deviation for the nearest neighbor matching and the 1:1 matching method were adopted using the MatchIt package of R (version 3.5.1; R Development Core Team).

## 3 RESULTS

### 3.1 Patients characteristics and Follow‐up information

This study included 3167 PTC patients, 1031 males (32.6%) and 2134 females (67.4%). The mean age was 41.6 years old with a range of 14–78, and 980 patients (30.9%) were over 55 years old. The clinical characteristics of patients were summarized in Table 1. The mean body mass index (BMI) was 23.9 with a range of 14.9–43.6. The mean size of the tumor was 0.87 cm: tumors >4 cm were found in 122 (3.9%) patients. Multifocality, bilaterality, capsular invasion were identified in 988 (31.2%), 679 (21.4%), 934 (29.5%) patients, respectively. Patients cohort were followed up regularly with a median follow‐up time of 42 months (range 8–72 months). During the follow‐up period, 68 patients (3.4%) in patients with LNM were diagnosed with local regional recurrence at a mean time of 27.8 months after the first surgery. Detailed information were shown in Table 2.

**TABLE 1:** Characteristics of the study patients (*N* = 3167).

Variable	*N*	%
Age, years, mean	41.6 (14–78)	
>55	980	30.9
BMI, mean	23.9 (14.9–44.6)	
<18.5	153	4.8
18.5–28	2592	81.8
>28	422	13.3
Gender		
Male	1033	32.6
Female	2134	67.4
Primary tumor size, cm^a^		
Mean	0.87 (0.05–6)	
>4 cm	122	3.9
Multifocal disease	988	31.2
Bilateral disease	679	21.4
Extrathyroidal extension	934	29.5

Abbreviation: BMI, body mass index.

^a^ The longest diameter of the largest lesion.

**TABLE 2:** Follow‐up information in different group of PTC patients.

Variable	CLNM (*n* = 1160)	LLNM (*n* = 388)	Skip (*n* = 73)	Total (*n* = 1581)
Follow‐up information				
Follow‐up time, month, median	40 (9–70)	42 (8–72)	40 (24–60)	42 (8–72)
Recurrences	34	32	2	68
Thyroid bed	1	3	0	4
Central LN regions	15	6	0	21
Lateral LN regions	18	22	2	42
Distant metastasis	0	1	0	1
Recurrence time^a^, month, mean	30.9 (9–60)	23.9 (8–48)	36 (32–40)	27.8 (8–60)

^a^ The time from surgery to first detection of recurrence.

### 3.2 Oncologic outcomes and detailed distribution of LNs

Contribution of 3167 patients T, N, and M to AJCC‐staging was shown in Table 3. LNM were found in 1581 (49.9%) patients. 1120 (35.4%) patients only had CLNM, which were defined as N1a. 388 (12.3%) with LLNM and 73 (2.3%) with skip metastasis constituted N1b. According to the 8th AJCC staging system, 2571 patients were categorized as stage I (81.2%), 486 as stage II (15.3%), 102 as stage III (3.2%), and 8 as stage IV (0.3%).

**TABLE 3:** Oncologic outcomes and LNs detail description.

Variable	*N*	%
TNM staging^a^		
T‐categories		
T1	2301	72.7
T2	296	10.4
T3	342	10.8
T4a	212	6.7
T4b	16	0.5
N‐categories		
N0	1586	50.1
N1a	1120	35.4
N1b^b^	461	14.6
Skip metastases	73	2.3
M‐categories		
M0	3164	99.9
M1	3	0.1
Stage I	2571	81.2
Stage II	486	15.3
Stage III	102	3.2
Stage IV	8	0.3
Lateral neck dissection		
No. of total LNs harvested, mean, LLNM/Skip	31.2 (21–56)/29.7 (17–49)	
No. of total LNs positive, mean, LLNM/Skip	9.37 (2–41)/3.51 (1–12)	
No. of central LNs harvested, mean, LLNM/Skip	8.33 (3–19)/8.21 (4–14)	
No. of central LNs positive, mean, LLNM/Skip	4.61 (1–13)/0	
Size of metastatic LNs foci, cm^c^, mean, LLNM/Skip	1.16 (0.3–3.1)/1.26 (0.2–3)	

Abbreviations: LN, lymph node; LNM, lymph node metastases; LLNM, lateral LNM.

^a^ 8th American Joint Committee on Cancer (AJCC) TNM staging system.

^b^ Including skip metastasis.

^c^ The longest diameter of the largest lesion.

Detailed distribution of LNs revealed that, of the patients with lateral lymph neck dissection, the mean numbers of total LNs positive/harvested in LLNM and skip metastasis patients were 9.37 (2–41)/31.2 (21–56) and 3.51 (1–12)/29.7 (17–49), respectively. More importantly, the mean numbers of central LNs positive/harvested in LLNM were 4.61 (1–13)/8.33 (3–19), whereas, those in skip metastasis patients were 0/8.21 (4–14). No significant difference in harvested central LNs number in these two groups revealed that comprehensive central LNs dissection is necessary to define skip metastasis. The mean size of the largest metastatic LN in LLNM and skip metastasis patients were 1.16 (0.3–3.1) and 1.26 (0.2–3) cm, respectively.

### 3.3 Surgical complications

Postoperative temporary and permanent hypocalcemia occurred in 445 (14.1%) and 22 (0.7%) patients, respectively. The mean parathyroid hormone (PTH) level of hypocalcemia patients was 5.79 (0.5–15.9) pg/ml, the mean PTH level of temporary hypocalcemia patients after 3 month was 33.2 pg/mL. 182 (5.7%) patients had varying degrees of hoarseness or cough after surgery: of these, 163 (5.1%) had temporary vocal fold paralysis and 19 (0.6%) had permanent vocal fold paralysis. In addition, 12 (0.4%) patients underwent secondary surgery due to postoperative bleeding, and 13 (0.4%) patients had chyle leakage. The risk of surgical complications was significantly different between thyroidectomy+CLND and LLND. Patients underwent neck radical dissection (LLND) had longer inpatient days, higher risk of temporary and permanent hypocalcemia, vocal fold paralysis, bleeding, and chyle leakage comparing to those underwent thyroidectomy+CLND. Since LLND procedure was performed in 73 skip LN metastases, incidences of complications were similar to other LLNM, and significantly higher than those in CLNM (Table S1).

### 3.4 Recurrence free survival in pN_1_ patients

**FIGURE 1:**
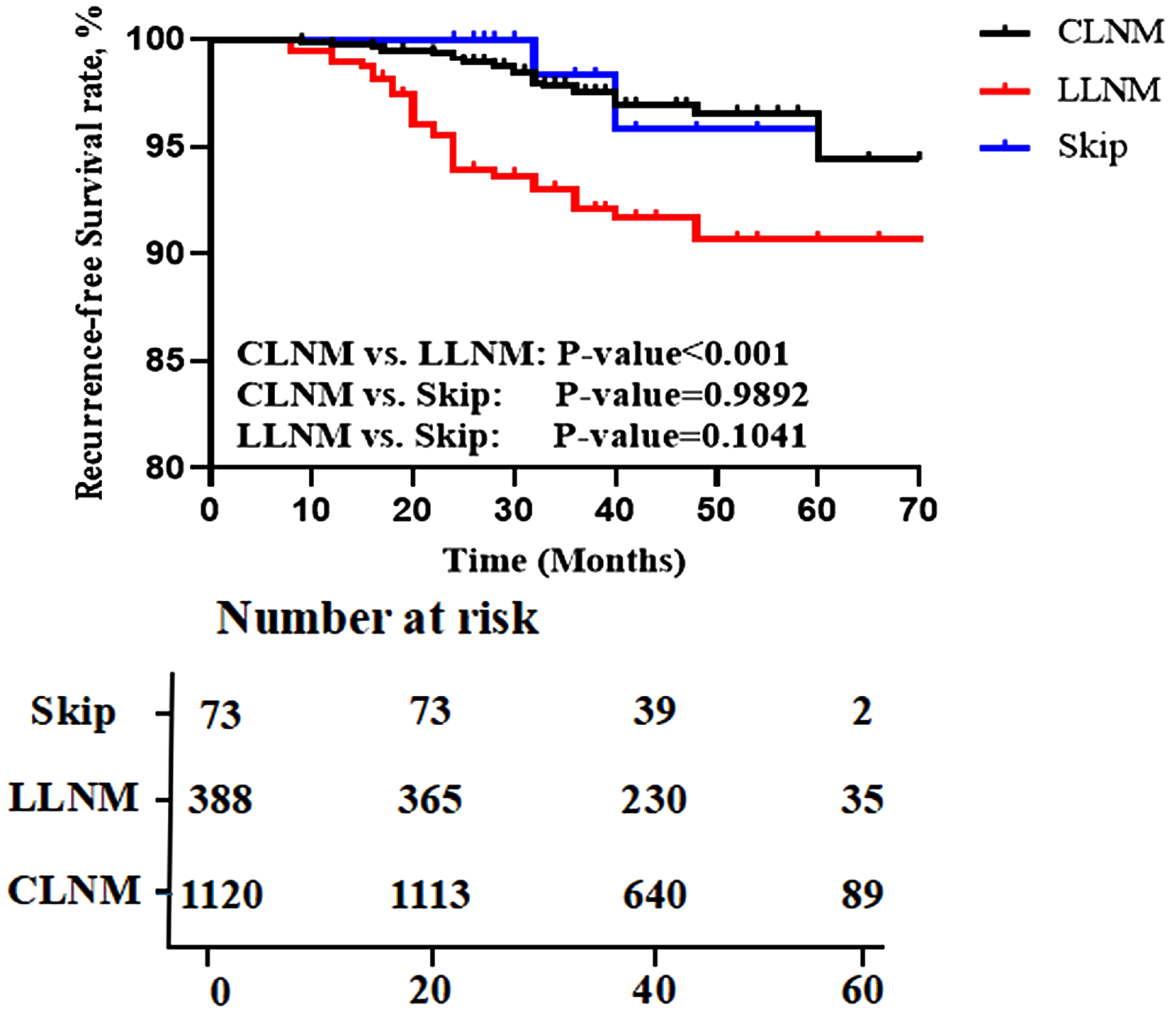
Kaplan–Meier plots of the RFS for PTC patients before propensity‐score matching.

During the period of follow‐up, 68 (4.3%) patients were diagnosed with recurrence in pN_1_ patients. The mean recurrence time was 27.8 (8–60) months after initial treatment. In detail, 34 (3.0%) cases recurred in 1120 CLNM (pN_1a_) patients. 32 (8.2%) cases recurred in 388 patients with LLNM and CLNM (pN_1b_), 2 (2.7%) cases recurred in 73 patients with skip metastasis (pSkip). In Figure 1 which show the recurrence‐free survival (RFS) at 3 years, significant difference was found between pN_1a_ and pN_1b_ patients (*p* value <0.001), as well as no statistically significant difference between pN_1b_ and pSkip patients (*p* value = 0.104). To standardize the enormous difference in the number of cases between pSkip and the other two groups, we used the method of propensity‐score matching to balance other clinical characteristics.

### 3.5 Recurrence free survival in pN_1_ patients after propensity‐score matching

**FIGURE 2:**
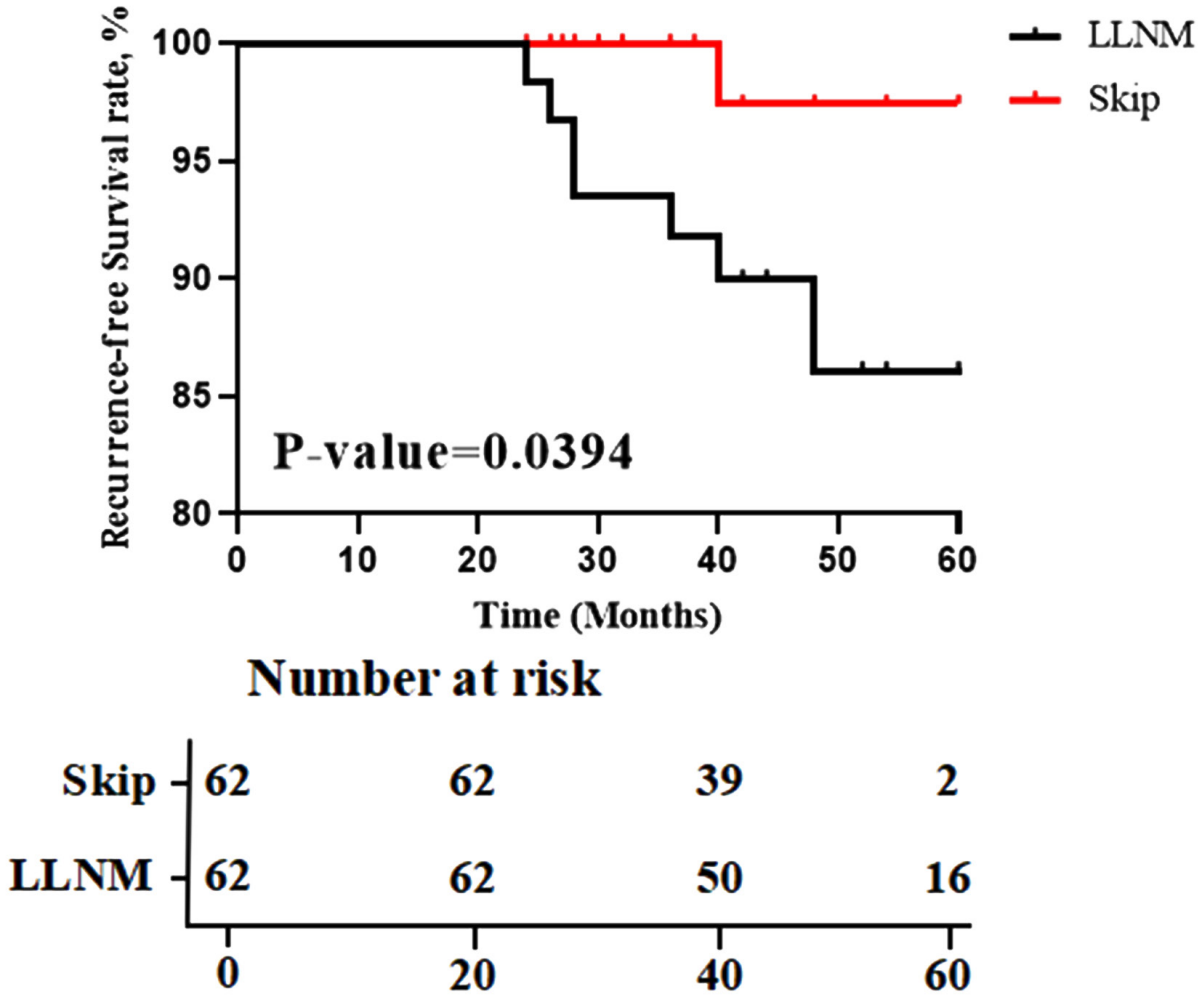
Kaplan–Meier plots of the RFS for matched cohort of patients with skip metastasis versus patients with LLNM (*n* = 62).

**FIGURE 3:**
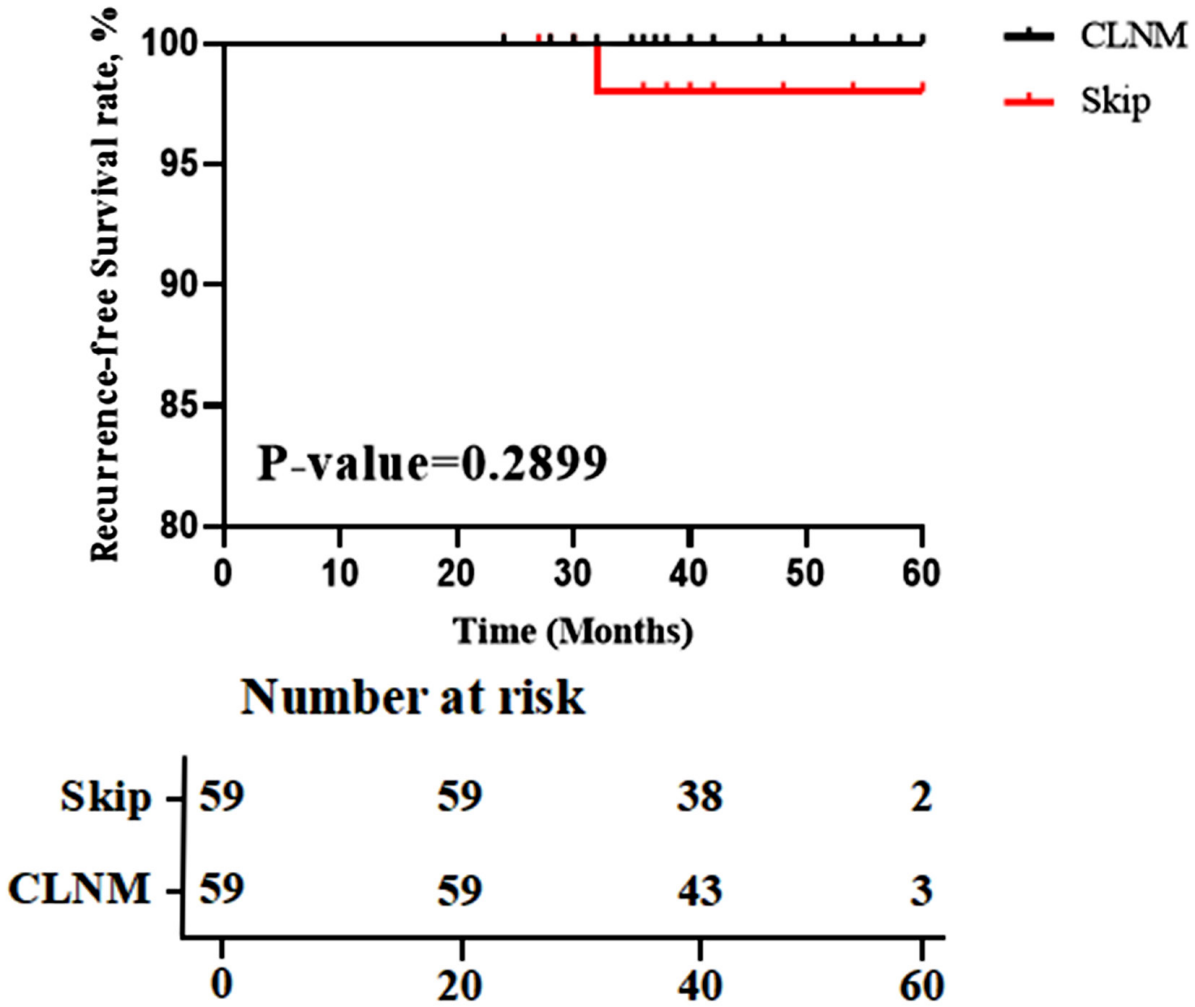
Kaplan–Meier plots of the RFS for matched cohort of patients with skip metastasis versus patients with CLNM (*n* = 59).

We created two propensity score‐matched cohorts by attempting to match pSkip patient with pN_1b_ patients (cohort 1), pSkip patient with pN_1a_ patients (cohort 2), respectively (a 1:1 match). After propensity‐score matching was completed, there were 62 matched pairs of patients in cohort 1 (Table S2) and 59 matched pairs of patients in cohort 2 (Table S3). The rate of recurrence risk in cohort 1 was 3.2% among pSkip patients and 25.8% among pN_1b_, while in cohort 2 was 3.4% among pSkip patients and 5.1% among pN_1a_. RFS at 60 months showed significant difference between pSkip and pN_1b_ patients (*p* value = 0.039), as well as no statistically significant difference between pSkip and pN_1a_ patients (*p* value = 0.29) (Figures 2 and 3).

## 4 DISCUSSION

Papillary thyroid cancer had standardized surgical strategy mode with recommended follow‐up and adjuvant treatment program. Given that PTC was an indolent disease associated with excellent survival overall, the ability to predict recurrence was a more meaningful outcome than to predict disease‐specific survival (DSS) for patients and clinicians. In contrast with predicting DSS, lymph node metastasis plays a more important role in assessing the risk of recurrence after initial treatment.^4^, ^22^ In this multicenter retrospective study, we found that the risk of recurrence for PTC patients with N1b metastasis was 8.8%, which was similar to the rates in other retrospective studies, significantly higher than that of patients with mere N1a metastasis.^4^, ^5^, ^6^, ^23^

The latest edition of AJCC/UICC TNM system downstaged a significant number of patients by removing regional lymph node metastases from the definition of T3 disease, which represented the N stage might play a limited role in predicting disease mortality. However, the staging system reserved the classification of the involved lymph nodes location (N1a vs. N1b), for the usefulness in assessing the risk of recurrence and early response to therapy.^13^, ^22^ Meanwhile, ATA Guideline, which was based on AJCC staging system, also required the designation of subsequent treatment regimens according to the characteristics of lymph node metastasis.^8^, ^24^ Researches including our retrospective study indicated that LLNM was associated with a high rate of posttreatment recurrence, which was increasingly considered an important factor associated with poor prognosis, high morbidity rates, and difficult reoperation. Moreover, LLNM usually required additional adjunctive therapy, including ^131^I, complete TSH suppressive therapy and elongated duration of follow‐up.^4^, ^25^, ^26^ Thus, lymph nodes metastases, including accurate evaluation and appropriate therapy, are still the key issue in the treatment of DTC at present.

Interestingly, the presence of skip metastasis, a special type of LLNM, played a different role in conventional LNM. The incidence of skip metastasis in LNM was relatively low, and the frequency was reported to vary between 6.8% and 21.8% in N1b patients.^12^, ^27^, ^28^, ^29^ Due to the low incidence of skip metastasis in PTC patients, only multicenter with large population of thyroidectomy could provide enough patient cohort, and few studies systematically focused on the therapeutic protocols and staging and recurrence of this kind of metastasis. The impact of skip metastasis on prognosis was also unclear. A systematic review comprehensively summarized the pathogenic mechanisms of skip metastasis and reinforces, indicating that skip metastasis did not affect patients' life expectancy.^30^ From more than 3000 PTC patients in this multicenter retrospective study, 73 patients with skip metastasis were pathologically confirmed, which has constituted the largest sample size of skip metastasis reported so far. In the present study, we studied on this topic from the perspective of recurrence, and verified the possible mechanism of skip metastasis by comparing the difference in recurrence between patients with skip metastasis and other types of LNM.

Surprisingly, the recurrence risk of skip metastasis was not consistent with that of N1b patients as expected. On the contrary, skip metastasis and N1a patients have similar recurrence risk, both of which much lower than the recurrence probability of N1b patients. Thus, skip metastasis had a different mechanism from that of conventional LLNM, which should not be roughly classified as the second‐tier LNM (N1b). Although skip metastasis was defined as N1b in the current staging system, the risk of recurrence, staging and treatment mode were inconsistent with those of conventional LLNM.

The exact cause of skip metastasis was not clear, of which the lymph drainage and biological behavior might be different from the common LLNM. Our previous studies indicated that skip metastases was not an accidental event in large samples, but a special type of lymph node metastases related to tumor location and specific lymphatic drainage pathways: factors including upper pole locations, capsular invasion/extracapsular spread (ETE), multifocality, tumor size <10 mm were considered to have a certain correlation with skip metastases, which was consistent with others findings.^25^, ^27^, ^28^ Among them, upper pole location was believed to be closely related to the occurrence of skip metastasis in several studies, involving level III nodes most frequently, which was explained by the lymphatic drainage system of thyroid gland.^28^ When skip metastasis occurred, tumor position weighted more than tumor size and other characteristics, malignancy of which might be different from that of conventional LLNM.

N1b patients, those with LLNM, total thyroidectomy (TT), and postoperative RAI and more strict TSH suppression therapy are still the standard therapy as recommended in all guidelines.^8^, ^31^ The disadvantages of these treatments, such as higher complication rate, life‐long loss of glandular function, poorer life quality, and increased medical expenditure, were covered and neglected by high recurrence rate. However, in our study, patients with skip metastasis, currently staging N1b, showed the similar clinical and prognostic characteristics as N1a patients.^4^, ^32^ The downstaging of skip metastasis may reveal more conservative treatment strategy, similar to the “less is more” treatment for PTC advocated in the 2015 ATA management guideline. In this scenario, unilateral PTC patients with highly suspected or intraoperative pathological diagnosed skip metastasis may benefit more from thyroid lobectomy (TL) with therapeutic compartmental lateral neck dissection instead of total thyroidectomy (TT).^33^, ^34^ Recent research showed that N1b patients with no evidence of CLNM might not require or benefit from prophylactic CND, which provided patients with skip metastasis a possibility of less aggressive treatment strategy.^35^, ^36^ In terms of adjunctive treatment, routine RAI and moderate or complete TSH‐suppressive therapy may not be necessary as well. Although the population of skip metastasis patients is not large, huge improvements in physiological, psychological, and social benefits are worthy. Thus, the N staging for skip metastasis could be potentially modified from N1b to N1a in aim to provide precise therapeutic maneuver for those patients.

The limitation in our current study was associated with retrospective design. In addition, our median follow‐up time was only 34 months, lacking of a long‐term follow‐up of PTC patients after surgery compared with other studies. However, researches showed a recurrence time of PTC was mostly within 36 months after initial treatment,^4^, ^5^, ^37^ which was consistent with our findings, indicating our analysis of a 3‐year recurrence rate of PTC patients in the same period was supported and reliable.

## 5 CONCLUSION

In conclusion, our study indicated that, among patients with LLNM, those with positive skip metastasis showed significantly lower recurrence, exhibiting a similar rucurrence tendency as patients with CLNM. Thus, skip metastasis could be categorized into N1a stage rather than N1b stage based on the AJCC TNM staging system. The downstaging of skip metastasis may reveal more conservative treatment strategy.

## AUTHOR CONTRIBUTIONS

**Zheyu Yang:** Conceptualization (equal); data curation (equal); formal analysis (equal); funding acquisition (equal); investigation (equal); methodology (equal); project administration (equal); supervision (equal); validation (equal); writing – original draft (equal); writing – review and editing (equal). **Yu Heng:** Conceptualization (equal); data curation (equal); formal analysis (equal); funding acquisition (equal); investigation (equal); methodology (equal); supervision (equal); validation (equal); visualization (equal); writing – original draft (equal); writing – review and editing (equal). **Qiwu Zhao:** Conceptualization (equal); data curation (equal); methodology (equal); writing – original draft (equal). **Hao Ding:** Conceptualization (equal); data curation (equal); supervision (equal); writing – original draft (equal). **Lei Tao:** Conceptualization (equal); data curation (equal); visualization (equal). **Xiaxing Deng:** Conceptualization (equal); data curation (equal); investigation (equal); methodology (equal); supervision (equal); writing – original draft (equal); writing – review and editing (equal). **Wei Cai:** Conceptualization (equal); data curation (equal); formal analysis (equal); funding acquisition (equal); investigation (equal); methodology (equal); supervision (equal); visualization (equal); writing – original draft (equal); writing – review and editing (equal). **Weihua Qiu:** Conceptualization (equal); data curation (equal); formal analysis (equal); funding acquisition (equal); investigation (equal); methodology (equal); project administration (equal); resources (equal); software (equal); supervision (equal); validation (equal); visualization (equal); writing – original draft (equal); writing – review and editing (equal).

## FUNDING INFORMATION

This research was supported by Shanghai Jiaotong University (YG2019ZDA15), and the National Natural Science Foundation of China (NSFC, 82072948).

## CONFLICT OF INTEREST STATEMENT

No conflict of interest exists in the research.

## Supporting information


Table S1

Table S2

Table S3

## Data Availability

The data that support the findings of this study are available from the corresponding author, Weihua Qiu, upon reasonable request.
